# Effects of Patient-Generated Health Data: Comparison of Two Versions of Long-Term Mobile Personal Health Record Usage Logs

**DOI:** 10.3390/healthcare10010053

**Published:** 2021-12-28

**Authors:** Do-Hoon Kim, Yura Lee, Ji Seon Oh, Dong-Woo Seo, Kye Hwa Lee, Young-Hak Kim, Woo Sung Kim, Jae-Ho Lee

**Affiliations:** 1 Department of Information Medicine, Asan Medical Center, University of Ulsan College of Medicine, Seoul 05505, Korea; k8016851@gmail.com (D.-H.K.); haepary@naver.com (Y.L.); doogie55@naver.com (J.S.O.); leiseo@gmail.com (D.-W.S.); geffa79@gmail.com (K.H.L.); mdyhkim@amc.seoul.kr (Y.-H.K.); wooskim1@gmail.com (W.S.K.); 2 Department of Emergency Medicine, Asan Medical Center, University of Ulsan College of Medicine, Seoul 05505, Korea; 3 Big Data Research Center, Asan Institute for Life Science, Asan Medical Center, Seoul 05505, Korea; 4 Department of Cardiology, Asan Medical Center, University of Ulsan College of Medicine, Seoul 05505, Korea; 5 Department of Pulmonary& Critical Care Medicine, University of Ulsan College of Medicine, Seoul 05505, Korea

**Keywords:** personal health records, mobile health, patient-generated health data, patient engagement

## Abstract

Patient-generated health data (PGHD) can be managed easily by a mobile personal health record (mPHR) and can increase patient engagement. This study investigated the effect of PGHD functions on mPHR usage. We collected usage log data from an mPHR app, My Chart in My Hand (MCMH), for seven years. We analyzed the number of accesses and trends for each menu by age and sex according to the version-up. Generalized estimating equation (GEE) analysis was used to determine the likelihood of continuous app usage according to the menus and version-up. The total number of users of each version were 15,357 and 51,553, respectively. Adult females under 50 years were the most prevalent user group (30.0%). The “My Chart” menu was the most accessed menu, and the total access count increased by ~10 times after the version-up. The “Health Management” menu designed for PGHD showed the largest degree of increase in its likelihood of continuous usage after the version-up (1.245; *p* < 0.0001) across menus (range: 0.925–1.050). Notably, improvement of PGHD management in adult females over 50 years is needed.

## 1. Introduction

Information technology advancements have enabled access to a variety of information anytime, anywhere, and can even create and distribute information through computers or mobile devices. In this context, the paradigm shift to patient-centered medicine has highlighted the importance of personal health records (PHR) [1,2,3]. Mobile PHRs (mPHRs) can collect and manage personal health information, and can be connected with other mobile services [4,5,6]. mPHR applications (apps) enable patients to access, monitor, record, and update their health information regardless of time and location [7]. Furthermore, mPHRs improve patients’ access to medical information, and their communication and relationships with medical teams. Consequently, patients can actively participate in their treatment to improve treatment compliance, efficiency, and quality. mPHR apps can provide evidence-based health information to users at a low cost [8]. In this way, mPHRs can have substantial impact on various disease-related health areas, including managing chronic diseases and mental health [4,8,9].

Patient engagement is essential in health care services and provides the greatest benefit to patients [4]. In precision medicine, patient engagement and patient-generated health data (PGHD) are considered as important as clinical information and genomic data. The information obtained through the mPHR can be helpful in knowing the patient’s personalized criteria [10]. However, there are concerns about the construction of mPHRs to obtain such PGHD [11,12]. mPHR apps for patients and medical staff are frequently discontinued and retired [4]. To fully utilize the collected information, inducing long-term usage by the user is needed. Only a handful of previous studies focused on the continued use of mPHR services [13,14,15,16]. One study reported that it is important for mobile app users to frequently enter data into the app to monitor health-related behaviors for effective weight loss, regardless of the region and app version [14]. Another researcher found that patients’ intention to continue using mPHR services was closely related to their regular use of self-monitoring features [13]. However, long-term studies of modifiable factors (e.g., service menus) and the persistence of mPHRs are lacking. Studies on modifiable factors affecting the duration of continuous use should be conducted considering the user’s unique characteristics like age and gender.

Here, we investigated the effect of PGHD functions on mPHR usage by comparing seven years of usage log data of an mPHR app distributed by a tertiary hospital in South Korea. The mPHR app, called My Chart in My Hand (MCMH), was first developed in 2010 and provides several functions for users to log their health data. A newer version of the app strengthened many functions including PGHD.

## 2. Materials and Methods

### 2.1. Development of the “My Chart in My Hand” Application

**Figure 1:**
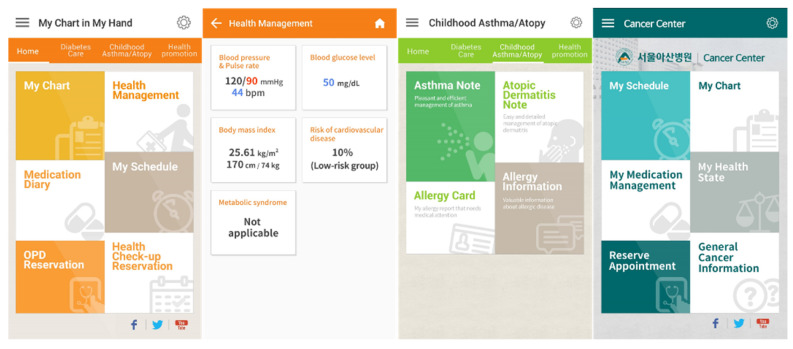
Screenshots of “Home” menu, “Health Management” menu, “Childhood Asthma/Atopy” menu, and “Cancer Center” from My Chart in My Hand version 2.0.

Asan Medical Center (Seoul, South Korea) is the largest tertiary hospital in the country with approximately 2700 registered inpatient beds and a large in-house hospital information system. In January 2011, the center developed and released MCMH 1.0 (Asan Medical Center, Seoul, South Korea), an Android operating system-based tethered mPHR app [17,18]. In the MCMH 1.0 study, it was concluded that it was necessary to expand services for chronically ill patients [18]. There were requests for PGHD, such as a symptom log and disease diary, at the relevant centers, reflecting the needs of the medical staff and patients. An iPhone-based MCMH was also necessary. In response to these needs, version 2.0 was developed. In December 2015, MCMH 1.0 was updated to MCMH 2.0, and iOS was added as an operating system. In MCMH 2.0, only registered patients can use the app, and it had additional patient engagement functions like disease diary, symptoms, lifestyle, quality of life, and stress for patients with cancer, diabetes, childhood asthma, and atopic dermatitis. MCMH 2.0 also provided functions like drug consultation with clinical pharmacists. The differences between the app versions are shown in Table 1. The “Health Management” menu is for patient-generated health data entered by the patient. Data in the “My Chart” menu are retrieved from the hospital information system. The data entered by the patient and the EHR data are stored in the hospital’s mobile server and EHR server. Figure 1 is composed of screenshots of MCMH version 2.0. Screenshots of MCMH version 1.0 can be found in the bibliography [18].

### 2.2. Study Design and Procedure

To identify MCMH’s usage pattern, we collected the usage log data spanning over seven years from January 2011 to May 2018; MCMH 1.0 (2.0) was used for 59 (28) months from January 2011 (February 2016) to November 2015 (May 2018). In the analysis, we only included the home menus of the MCMH, which included “Health Management”, “My Chart”, “Today’s Medication”, and “Online Appointment”.

**Figure 2:**
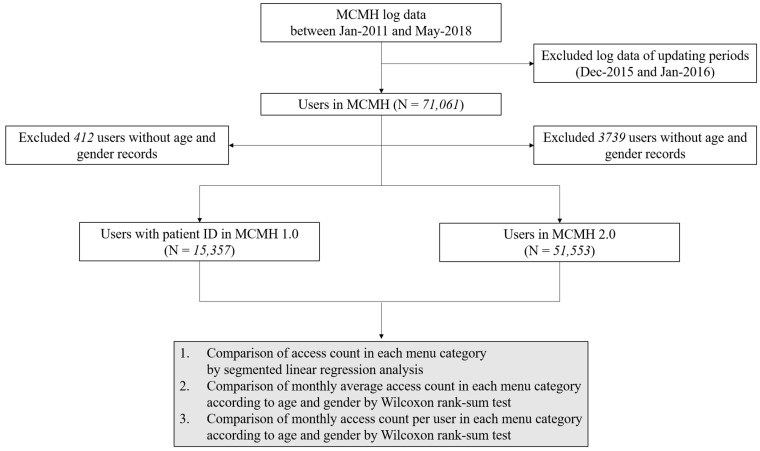
Study flow diagram. MCMH: My Chart in My Hand, ID: identifier.

We compared the monthly average access count and monthly access count per user in each menu in MCMH 1.0 and MCMH 2.0 according to age and sex. The user group was divided into children and adolescents (below 19 years old), females under 50 (19 to 49 years old), males under 50 (19 to 49 years old), females over 50 (50 years or older), and males over 50 (50 years or older). The age of 50 was used to divide the adult user group considering that the prevalence of chronic diseases increases after that age. Figure 2 shows the study flow.

This study was approved by the institutional review board of Asan Medical Center (no. 2018-0321). The need for informed consent was waived as we used routinely collected log data that were managed anonymously at all stages, including during data cleaning and statistical analyses.

### 2.3. Statistical Method

The longitudinal effects, according to the version-up (i.e., MCMH 1.0 → MCMH 2.0), on the overall usage trend of the MCMH app were analyzed using a two-phase interrupted time series design with segmented linear regression analysis. To avoid possible bias caused by the transfer of existing users after the version-up, the usage log data of two months prior to the version-up (i.e., December 2015 and January 2016) were excluded from the analysis. The change in slope was compared with the slope ratio before and after the version-up. The monthly average access count per user, defined as each patient’s access count of each menu every month, was compared between MCMH 1.0 and MCMH 2.0 using the Wilcoxon rank-sum test.

We analyzed the log data using the generalized estimating equation (GEE) to predict the probability of logging in in the next week according to menu usage in this week. Login was defined as the presence or absence of logging in to MCMH in the next week where the patient was assigned. The menu used was defined as the presence or absence of each menu’s usage this week. All users were considered to be discontinued when the app was not used for 24 weeks (6 months), and their data were excluded after discontinuation. Furthermore, we excluded users who used it only once. Using the GEE analysis, we also evaluated the interaction effect to see if there was a difference between versions.

All analyses were carried out in R software version 3.5.3 (R Foundation for Statistical Computing, Vienna, Austria) and the GEE analysis in SAS (version for 9.4). All P values less than 0.05 were considered statistically significant.

## 3. Results

### 3.1. User Characteristics

**Figure A1:**
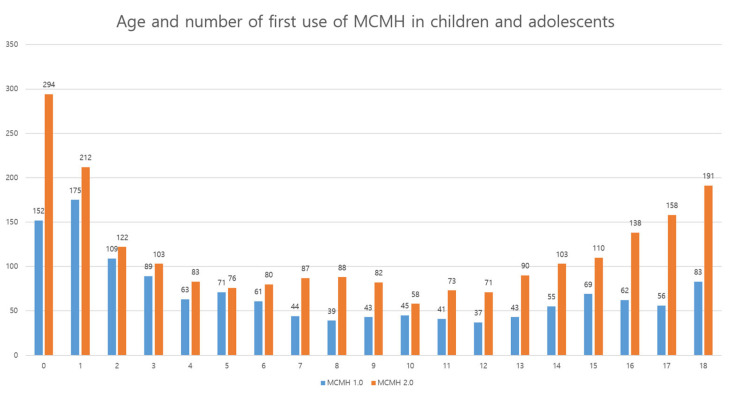
Age and number of first use of MCMH in children and adolescents.

The total number of users of MCMH 1.0 and MCMH 2.0 were 15,357 and 51,553, respectively (Table 2). The proportion of males was higher than females in both MCMH 1.0 (54.5% versus 45.5%) and MCMH 2.0 (50.4% versus 49.6%). Females under 50 were the most prevalent user group in both MCMH 1.0 (29.3%) and MCMH 2.0 (30.3%). The age of first use by children and adolescents was mainly in the 0–3-year-old category, which was similar in MCMH 1.0 and 2.0. The ages of children and adolescents who used the app for the first time are described and presented in Figure A1.

### 3.2. Overall Usage Trend

The login counts of MCMH 1.0 and MCMH 2.0 were 849,134 and 3,672,568, respectively (Table 3); the access counts notably increased after the version-up. The most commonly accessed menu was “My Chart,” whose total number of access counts steeply increased by approximately 10 times after the version-up. In the “My Chart” menu, the most visited submenu was “Laboratory Results,” whose access count per month also steeply increased after the version-up (54,453 versus 559,071) (Table 3). The fold increases in the access counts of “Health Management” (4.68) and “Today’s Medication” (2.57) were smaller than that of total logins (8.96), whereas the access count of “Online Appointment” (7.18) was more comparable to that of total logins.

**Figure 3:**
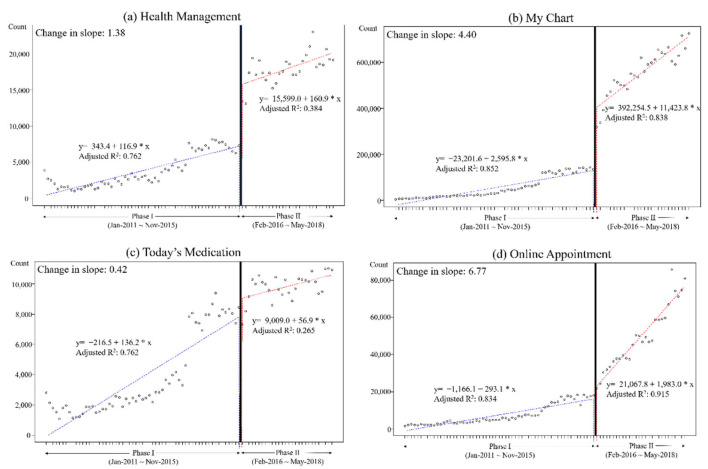
Segmented regression plots of the monthly access counts of (**a**) “Health Management” menu, (**b**) “My Chart” menu, (**c**) “Today’s Medication” menu, and (**d**) “Online Appointment” menu.

The slopes of the monthly access count in all menus except “Today’s Medication” increased after the version-up (Figure 3). The slope of the “Health Management” menu increased by 1.38 times from 116.9 to 160.9. The slope of the “My Chart” menu increased by 4.40 times from 2595.8 to 11423.8. The slope of the “Online Appointment” menu increased by 6.77 times from 293.1 to 1983.0, which was the highest rate of increase among the menus. However, the slope of the “Today’s Medication” menu decreased by 0.42 times from 136.2 to 56.9.

### 3.3. Monthly Average Access Count

**Figure 4:**
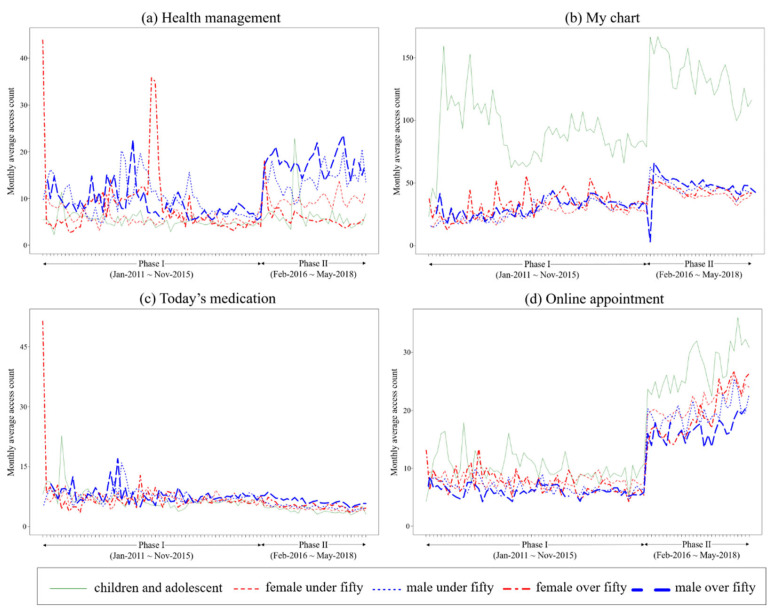
Monthly average access counts of (**a**) “Health Management” menu, (**b**) “My Chart” menu, (**c**) “Today’s Medication” menu, and (**d**) “Online Appointment” menu according to age and sex groups.

The median values of the average monthly access counts are shown in Table 4, and the changes in these counts over time are shown in Figure 4. The median value of average monthly access counts for the “Health Management” menu increased from 6.4 to 9.5. The increases were statistically significant for females under 50, males under 50, and males 50 years or older. The median value for the “My Chart” menu increased from 31.6 to 46.5. The value was the highest in children and adolescents in both MCMH 1.0 and MCMH 2.0 (89.7 and 134.6, respectively). The median values for the “Today’s Medication” menu significantly decreased in all groups between MCMH 1.0 and MCMH 2.0. The median value for the “Online Appointment” menu increased from 7.2 to 19.9; again, the value was the highest in children and adolescents in both MCMH 1.0 and MCMH 2.0 (9.8 and 26.1, respectively).

### 3.4. Monthly Access Count per User

**Figure 5:**
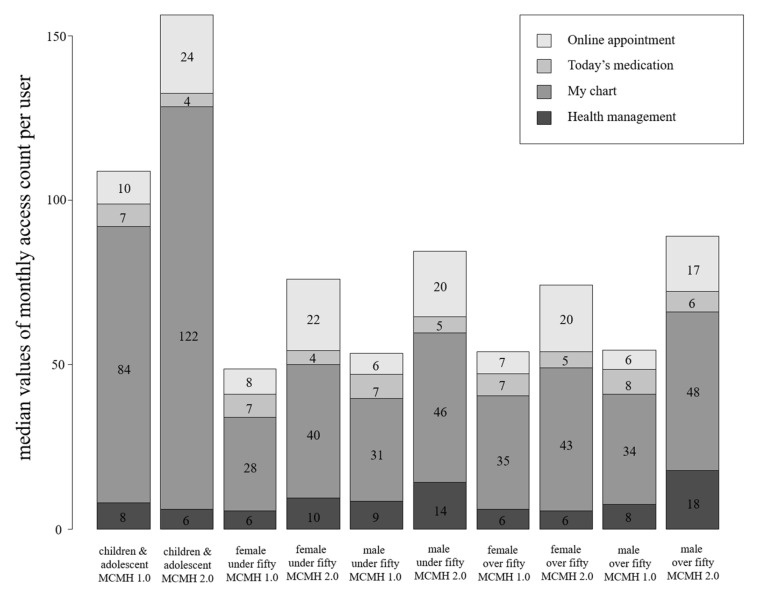
Bar plot of median values of monthly access count per user in MCMH 1.0 and MCMH 2.0 according to age and sex groups.

The median values of the monthly access count per user are shown in Table 5 and Figure 5. MCMH’s total usage increased as the number of users increased upon the version-up. Overall, the monthly access count per user increased in the “My Chart” and “Online Appointment” menus, but not in the “Health Management” and “Today’s Medication” menus. 

The median value of monthly access count per user in the “My Chart” menu increased from 15 to 16; particularly, it was the highest in children and adolescents in both MCMH 1.0 and MCMH 2.0 (27 and 29, respectively). In the “Online Appointment” menu, the median value of monthly access count per user increased from 3 to 8; particularly, it was the highest in children and adolescents in both MCMH 1.0 and MCMH 2.0 (4 and 10, respectively). 

The median values of monthly access per user in “Today’s Medication” and “Health Management” significantly decreased in all groups between MCMH 1.0 and MCMH 2.0, although in the “Health Management” menu, it was not significantly different in males over 50 (*p* = 0.115). This result may be because of the relatively decreased median value as the number of users of MCMH 2.0 increased rapidly.

### 3.5. Changes in Functions’ Usage in MCMH According to Version-Up

The effect of the next week’s login on the use of the “Health Management” menu by version had a statistically significant difference (Table 6). This menu was designed for management of PGHD. Compared to MCMH 1.0, the effect of this menu in MCMH 2.0 is 1.245 times larger. When considering version and interaction, the odds ratio of this menu was the highest among the used menus. In the data including MCMH 1.0 and MCMH 2.0, the odds ratio that a person who used the “Online Appointment” menu would login next week increase significantly to 1.328 times after the version-up. Further, the odds of users using the “My Chart” menu logging in next week increased significantly to 1.288 times. However, considering version and interaction, the effect of “Online Appointment” menu decreased by 0.925 times in MCMH 2.0 compared to MCMH 1.0, while the effect was not high in “My Chart” menu at 1.040 times.

## 4. Discussion

### 4.1. Principal Findings

We analyzed seven years of usage logs of an mPHR in a large-sized hospital and compared them before and after the app’s version-up. Although the version was up, the “Health Management” menu where patients with PGHD actively participate was important. Among the menus used when considering the version and interaction, the “Health Management” menu had the highest odds ratio. These results suggest that the importance of allowing users to continue to use the mPHR app to enter and monitor user health data has increased over time. This is similar to the findings of the previous study [13]; however, it was proven to be valid with long-term use, over a seven-year period, in a larger number of patients, even when the version was changed. We also found that the usage pattern of mobile apps differs based on gender and age group.

According to a recent study, patient engagement has become more important in the study of continued use of mobile apps. Lee et al. found that the tendency of a declining mHealth service use rate was alleviated in patients who used the self-monitoring function stating that this tendency had a positive effect on continued use [13]. However, this was a relatively small sample of approximately 1500 individuals over a relatively short study period of 18 months. Han et al. found that the data entry frequency of mobile apps was significantly associated with weight loss over time [14], but only had a relatively short-term effect over a 12-month study period. Our study also considered an upgraded version over a long period of seven years and analyzed a relatively large number of users (over 5000). We hope that our research will contribute to the understanding of the sustained use of PHR and help improve the design and delivery of consumer-centric medical technologies.

### 4.2. Usage Trends of the MCMH According to Version-Up

mPHRs enable patients to access health information via the internet or telecommunication devices like cellular phones and tablet computers [19], thereby allowing more patients to engage with their own healthcare data more often and in a more timely manner [20]. Currently, there are more than 250,000 mPHR apps for public use that are available for download [21,22]. Accordingly, the number of registered users and their usage have grown steadily [23,24,25], especially for chronic diseases [20]. In our study, MCMH logins were frequent and increased substantially during the study period. In addition, the access count increased significantly after the version-up.

### 4.3. Usage Pattern

The most popular menu in terms of total access count and the average count per month was the “My Chart” menu in both MCMH 1.0 and MCMH 2.0, with the access counts showing notable increases after the version-up. Among the functions of the “My Chart” menu, the types of tests provided in laboratory results were diversified in MCMH 2.0. In the case of Kaiser Permanente, the most visited features of their My Health Manager PHR in 2007 were lab test results and the registrations increased rapidly including online test results [25]. In this study, laboratory results are found within the “My chart” menu and were the most accessed menu in both MCMH 1.0 and MCMH 2.0. When upgrading an app, one needs to consider which function should be improved that patients may use. Users respond to informative and new features. Even for a highly useful function, the overall usage may not show significant changes if there are no significant changes in the version-up, as in the case of the “Today’s Medication” menu in our study.

### 4.4. Monthly Average Access Count and Monthly Access Count per User

We found that the monthly average access count and its changes over time varied according to age and sex. For the “My Chart” menu, the average usage was significantly higher in children and adolescents than in other groups, and increased significantly after the version-up. Similarly, Ketterer et al. reported that the most frequently accessed feature in a patient portal system of a pediatric primary care population was the lab results [26]. We presume that the amount of mPHR use by parents and care providers for the children and adolescents was greater than the amount directly used by children or adolescents. In a previous study, patients in the 0–19 age group used the app more frequently among users with patient IDs [18]. These findings may be due to parents accessing MCMH on behalf of younger patients, particularly children [18]. According to surveys, the wait time for new appointments increased by 30% between 2014 and 2017 [27]. Thus, the needs of patients regarding appointments are expected to increase. In this study, the use of the “Online Appointment” menu increased across all groups and showed the highest degree of increase among all menus. Therefore, it may be necessary to add the functionality of “Online Appointment” tailored to the needs of the users of mPHR apps.

### 4.5. Effect of PGHD in mPHR

In females, the number of users and the access count of the “Health Management” menu varied according to age; the number of female users over 50 was notably less than those under 50 and they showed few access counts. The access count for this menu did not increase after the version-up in children and adolescents and females over 50. Jung and Padman reported that female patients were more actively engaged in their health management [28]. We also observed that the “Health Management” menu’s usage increased in females under 50 but not in those over 50. Wildenbos et al. showed that the use of mPHRs by older adult patients aged 50 years and above may be hindered due to the lack of thoughtful user interface designs that help overcome the age barriers to technology. While using the app, the benefits of the new technology were not obvious to the elderly, which led to frustration and a desire to stop using it [29]. Other studies have suggested that the benefits of new technology should be readily apparent while using it as the elderly population uses it [30,31]. Therefore, the health management menus should be further improved to consider the age barriers in technology. Furthermore, it may be helpful to provide patient engagement menus that are aimed at children and adolescents.

### 4.6. Limitations of the Study

There are some limitations to this study. The results may be limited in generalizability because the data were obtained from a single hospital. Moreover, we did not analyze other possible factors that may affect the mPHR’s usage, including socio-economic status, time required for hospital visits, encouragement by physicians and family attendance, technical anxiety, ease of use, and health issues, among others. Another limitation is that the analysis did not include the app features added in the version-up that may have contributed to increased app usage. For example, the disease diary function in the “Health Management” menu was not included in the analysis because it was newly added after the version-up. Further, our study did not compare all menus that are common in MCMH 1.0 and MCMH 2.0. Lastly, the number of users of MCMH 2.0 increased significantly compared to MCMH 1.0, but there is no way to quantitatively evaluate it. Various variables may be involved in this increase, like social mobile health interest, mPHR app promotions, and addition of app support for iOS. Nevertheless, our methods for analyzing the differences in the mPHR functions’ usage according to users may be generalizable. Furthermore, other mPHR or mobile health services should be pursued to improve patient compliance and outcomes by providing customized mPHR functions.

## 5. Conclusions

mPHR app usage has increased over time. A major update accelerated this trend and caused the largest increase in likelihood of continuous usage for the menu designed for PGHD. However, improvements in PGHD management for females over 50 years is needed. Managing PGHD is important for users’ continued app usage. Studies on the clinical utility of PGHD, methods of improving user interfaces, and the effects of connections with wearable devices are needed.

## Figures and Tables

**Table 1:** Differences in the features between MCMH ^1^ 1.0 and MCMH ^1^ 2.0.

	MCMH ^1^ 1.0	MCMH ^1^ 2.0
Operating system	Android	Android + iOS
User	Available to the general public	Only patients registered in the hospital
Chronic disease management	Primitive	Cancer, diabetes, pediatric asthma/atopic dermatitis, health promotion
Key values	Patient empowerment (access to medical information)	Patient engagement (diary, survey)
OPD ^2^ support	OPD ^2^ waiting list	Information and guide, my schedule, payment
Changes during the version-up	− Newly created: “Survey” menu (ulcerative colitis, Crohn’s disease, and Bechet colitis) − Newly created: “Waiting Screening” submenu − “Height” and “Weight” submenus were integrated into the “Body Mass Index” submenu − “Insulin Medication History Management” submenu was integrated into the “Diabetes Management” menu − All menus were divided into the history list, history graph, registration, modification, and detailed screen	

^1^ My Chart in My Hand. ^2^ Outpatient department.

**Table 2:** Demographic profiles of the users of My Chart in My Hand (MCMH) according to age and sex.

	MCMH 1.0 (59 Months)	MCMH 2.0 (28 Months)	Total (87 Months)	*p*-Value
Users, n	15,357	51,553	66,910	-
Age, years (mean ± standard deviation)	41.7 ± 16.7	45.6 ± 15.6	45.0 ± 16.0	<0.001
Sex, n (%)	-	-	-	<0.001
Male	8364 (54.5)	25,961 (50.4)	34,325 (51.3)	-
Female	6993 (45.5)	25,592 (49.6)	32,585 (48.7)	-
Group, n (%)	-	-	-	<0.001
Children and adolescents (<19 years)	1337 (8.7)	2219 (4.3)	3556 (5.3)	-
Females under 50 (19–50 years)	4494 (29.3)	15,604 (30.3)	20,098 (30.0)	-
Males under 50 (19–50 years)	4148 (27.0)	11,780 (22.9)	15,928 (23.8)	-
Females over 50 (50 years or older)	1895 (12.3)	8972 (17.4)	10,867 (16.2)	-
Males over 50 (50 years or older)	3483 (22.7)	12,978 (25.2)	16,461 (24.6)	-

**Table 3:** Total access count and access count per month of the functions in My Chart in My Hand (MCMH).

	MCMH 1.0		MCMH 2.0		Fold Increase
	Total Access Count	Access Count /Month	Total Access Count	Access Count /Month	
Login	849,134	14,640	3,672,568	131,163	8.96
Functions	4,240,403	73,110	18,518,700	661,382	9.05
Health management	233,953	4034	529,139	18,898	4.68
Blood sugar test	68,532	1182	203,499	7268	6.15
Blood pressure	39,738	685	194,464	6945	10.14
Body mass index	78,003	1345	74,369	2656	1.97
10 CVD ^1^ risk	26,019	449	32,554	1163	2.59
Metabolic syndrome	21,661	373	24,253	866	2.32
My chart	3,352,838	57,808	16,248,385	580,300	10.04
Laboratory results	3,158,288	54,453	15,653,989	559,071	10.27
Condition	148,618	2562	352,642	12,594	4.92
Allergies	45,932	792	241,754	8634	10.92
Today’s medication	235,483	4060	292,180	10,435	2.57
Online appointment	418,129	7209	1,448,996	51,750	7.18

^1^ Cardiovascular disease.

**Table 4:** Median values of monthly average access counts in MCMH 1.0 and MCMH 2.0 according to age and sex groups.

Groups	Health Management			My Chart			Today’s Medication			Online Appointment		
	MCMH 1.0	MCMH 2.0	*p*-Value	MCMH 1.0	MCMH 2.0	*p*-Value	MCMH 1.0	MCMH 2.0	*p*-Value	MCMH 1.0	MCMH 2.0	*p*-Value
Children and adolescents	5.0 (4.5–5.8)	5.7 (4.6–6.7)	0.087	89.7 (78.6–104.4)	134.6 (124.0–149.3)	<0.001	6.3 (5.7–7.2)	3.9 (3.4–4.6)	<0.001	9.8 (8.7–11.4)	26.1 (24.6–30.4)	<0.001
Females under 50	5.6 (4.8–6.5)	9.4 (8.7–10.5)	<0.001	26.3 (22.3–28.7)	41.1 (38.4–44.3)	<0.001	6.8 (6.1–7.7)	4.3 (4.0–4.5)	<0.001	7.8 (7.2–8.4)	21.1 (19.4–22.8)	<0.001
Males under 50	8.6 (7.1–11.0)	13.9 (12.6–15.2)	<0.001	28.3 (10.7–33.7)	45.2 (43.3–49.3)	<0.001	7.5 (6.6–8.4)	4.8 (4.6–5.2)	<0.001	6.4 (5.9–7.0)	19.3 (18.5–20.5)	<0.001
Females over 50	5.8 (4.4–9.9)	5.3 (4.8–6.0)	0.381	32.5 (26.6–37.1)	44.4 (41.2–46.4)	<0.001	6.5 (5.9–7.5)	5.1 (4.8–5.5)	0.006	7.1 (6.0–8.4)	18.1 (16.1–23.0)	<0.001
Males over 50	7.9 (6.8–10.0)	17.7 (16.6–19.0)	<0.001	30.3 (24.6–34.5)	48.0 (46.5–51.9)	<0.001	7.7 (6.6–8.4)	6.2 (5.8–7.0)	<0.001	6.0 (5.4–6.6)	16.3 (15.6–17.9)	<0.001
Total	6.4 (5.0–9.1)	9.5 (5.9–14.9)	<0.001	31.6 (24.8–41.3)	46.5 (42.0–53.5)	<0.001	6.9 (6.1–8.0)	4.8 (4.2–5.5)	<0.001	7.2 (6.1–8.6)	19.9 (17.7–23.7)	<0.001

Values are shown as median (interquartile range). *p*-values were calculated by the Wilcoxon rank-sum test.

**Table 5:** Monthly access count per user in MCMH 1.0 and MCMH 2.0 according to age and sex groups.

Groups	Health Management			My Chart			Today’s Medication			Online Appointment		
	MCMH 1.0	MCMH 2.0	*p*-Value	MCMH 1.0		MCMH 1.0	MCMH 2.0	*p*-Value	MCMH 1.0		MCMH 1.0	MCMH 2.0
Children and adolescents	3 (1–7)	3 (1–6)	<0.001	27 (8–88)	Children and adolescents	3 (1–7)	3 (1–6)	<0.001	27 (8–88)	Children and adolescents	3 (1–7)	3 (1–6)
Females under 50	3 (1–6)	2 (1–5)	<0.001	13 (5–31)	Females under 50	3 (1–6)	2 (1–5)	<0.001	13 (5–31)	Females under 50	3 (1–6)	2 (1–5)
Males under 50	3 (1–7)	3 (1–6)	<0.001	13 (5–32)	Males under 50	3 (1–7)	3 (1–6)	<0.001	13 (5–32)	Males under 50	3 (1–7)	3 (1–6)
Females over 50	2 (1–5)	2 (1–4)	<0.001	15 (5–35)	Females over 50	2 (1–5)	2 (1–4)	<0.001	15 (5–35)	Females over 50	2 (1–5)	2 (1–4)
Males over 50	3 (1–6)	3 (1–6)	0.115	15 (6–35)	Males over 50	3 (1–6)	3 (1–6)	0.115	15 (6–35)	Males over 50	3 (1–6)	3 (1–6)
Total	3 (1–6)	2 (1–5)	<0.001	15 (6–37)	Total	3 (1–6)	2 (1–5)	<0.001	15 (6–37)	Total	3 (1–6)	2 (1–5)

Values are shown as median (interquartile range). *p*-values were calculated by the Wilcoxon rank-sum test.

**Table 6:** Generalized estimating equation analysis including interaction effect according to menu effect and version.

		My Chart in My Hand			
		Odds Ratio	95 % CI ^1^		*p* Value
			Lower	Upper	
Version		0.726	0.713	0.740	<0.001
Age		0.998	0.998	0.999	<0.001
Gender	Male	1.011	0.994	1.029	0.203
	Female	1.000	-	-	-
HM ^2^		1.134	1.092	1.178	<0.001
Version HM ^2^		1.245	1.188	1.306	<0.001
MC ^3^		1.288	1.252	1.325	<0.001
Version * MC ^3^		1.040	1.010	1.072	0.0093
TM ^4^		1.186	1.152	1.222	<0.001
Version * TM ^4^		1.050	1.014	1.087	0.0058
OA ^5^		1.328	1.296	1.361	<0.001
Version * OA ^5^		0.925	0.898	0.953	<0.001

^1^ Confidence interval. ^2^ Health Management. ^3^ My Chart. ^4^ Today’s Medication. ^5^ Online Appointment. * Analysis considering the interaction between version and each menu.

## Data Availability

Mobile phone data are proprietary and confidential. We obtained access to these data from the Research Information Unit of Asan Medical Center within the research project (IRB number: 2018-0321). For legal reasons, the full dataset cannot be published. However, access to mobility data for research purposes can be requested from the Research Information Unit of Asan Medical Center, Seoul.
